# Correlation between permanent tooth eruption pattern and the predominance of the motor function laterality

**DOI:** 10.4317/medoral.19567

**Published:** 2014-03-08

**Authors:** Ana Veloso-Durán, Carmen M. Vazquez-Salceda, Julian López-Jiménez, Margarita Veloso-Durán, Andreu Puigdollers

**Affiliations:** 1 DDS, Collaborator at the Hospital Nen Deu in Barcelona; 2 MD, DDS, MS, PhD, Associate Professor of Orthodontics and Integrated child pathology at the Faculty of Dentistry. University of Barcelona; 3 MD. Medical Resident of Traumatology at Hospital Mutua de Terrassa; 4 MD, DDS, MS, PhD, Associate Professor and Director of the Department of Orthodontics and Dentofacial Orthopedics, Universitat Internacional de Catalunya

## Abstract

Objectives: To asses whether dental eruption order can play a role in the early diagnosis of crossed laterality. Study Design: Dental eruption pattern along with eye, ear, hand and foot lateralism were examined on 131 children between 6 to 8 years old from public schools from a multietnic population area of Barcelona city. Statistic methods (Statgraphics Plus 5.1 program) were used to evaluate data recollected. Results: Only foot and dentition lateralities behave as independent variables regarding hand laterality. So dental eruption laterality (along with the foot one) would be one of the parameters more related to hand laterality given that dentition variable relationship is greater that the foot one. This suggests that tooth eruption could be more clinically relevant. Crossed laterality hand-foot is significantly more predominant in men (13%) than in women (1,6%). Meanwhile, the relationship between hand and dentition didn’t show any influence of sex. Conclusions: Dental eruption order, can be used as a good parameter in the determination of the patient’s laterality.

** Key words:**Dentition, dental eruption, motor laterality, crossed laterality.

## Introduction

One of the most challenging educative issues nowadays is academic failure. Teachers and educators have often wrongly labelled children who suffer from it as “clumsy” or “lazy”, even assuming intellectual coefficients below the normal range. Nonetheless, many causes lie beneath academic failure. Among the most frequent are specific learning disabilities, which affect a 15% of school-aged children and can be frequently caused by distortion in the gathering and interpretation of the information (1-3).

It is possible to help these children who suffer from this condition if it is detected and treated promptly, aiming to avoid consequential behavioural and self-steam issues.

There are a variety of opinions regarding the multiple causes of learning difficulties, some of which point out the possible role of an altered brain laterality, theory that still lacks enough scientific validity (1,4). This theory postulates that in cases with an abnormal lateralization an insufficient cerebral asymmetry would occur and this could interfere proper learning in different fields (whether or not combined with behavioural disorders) (2,3); although this concept also has detractors.

Nevertheless, when a family consults on these disorders, crossed laterality is a constant finding in the anamnesis as a crucial fact on the subject’s clinical history. That brings the spotlight to laterality of the dental eruption, given that the age in which children begin the dental replacement (around 6 years old) (5) coincides with laterality development. There seems to be a coincidence or correlation between first tooth replacement and motor laterality of the child, so that if it debuts or it’s more premature in the right hemiarcades, the child tends to be right-handed. On the contrary, when replacement starts in the left hemiarcades, tends to be left-handed (6).

The awareness and care of development problems in childhood is a task that involves a variety of specialists: medical doctors (pediatricians), dentists, psychologists and educators. The fact of proving the link between dental replacement and motor laterality can be useful in early detection of problems arising from crossed laterality. It would contribute on childhood psychology, paediatrics and odontology in an easy yet effective way. Literature shows only partial studies on this subject (6-8).

The purpose of this study has been to asses whether dentition can play a role in the diagnosis of crossed laterality thorough an analysis on a significant number of children at the proper age with the appropriate statistic tools. This may provide the different professionals involved in the pathology with an early diagnosis tool that can help them to get a more individualized and thus adequate therapy.

## Material and Methods

Subjects: The subjects of this study were children from both sexes coming from primary education public schools belonging to high multietnic sanitary area in Barcelona city. Data collection was carried out during the mandatory oral revisions established for these schools, which were added crossed laterality tests. The children attended first, second and third grade of primary school (ages ranged from 6 to 8 years old). All the students that fulfilled the inclusion criteria from the classes selected were studied. The inclusion criteria included all the healthy children in the age rank. Healthy was defined as lack of an associated pathology that might interfere with the results of the tests, so children with other motor limitations and behavioural or mental disorders were excluded. Finally, a total of 130 children were studied, with an mean age of 6,723 years and a range of age between 6 and 8, divided in 53,08% girls and a 46,92% boys.

Methods: Our work took two parts: a first one about study and exploration of the dentition pattern and a second one about a motor laterality evaluation. At the dentition pattern study, it was carried out an oral exploration to determine the kind of dentition (temporal or mixed) and the order of eruption, comparing both sides (8,9). Laterality in dentition was defined by a more premature eruption of central and lateral incisors, and firsts permanent molars, in left or in right hemiarcades (both superior and inferior). When there was no dominance coincidence between maxilar and mandibular teeth the patient was awarded to the more erupted teeth. In the second time, laterality on hands, feet and hearing was evaluated through different methods (1).

The dominance of one body side over the other (motor laterality) was determined using criteria established by different specialists in child pedagogy and psychology fields (5,10,11). For hand evaluation it was used actions as throw or collect a ball from the floor, erase with a rubber, wrinkle a paper, brush teeth, comb hair, pick up a glass and insert objects in a box. For foot measurements it was used actions as kick a goal, drag a rubber along the floor, get a ball from underneath the table and hit with the foot. Eye assessment was made by looking through a hole in a sheet, a tube and a keyhole. Finally, assessment of ear laterality was done by listening to a cellular phone, a phrase in lower voice, approaching the ear to a door and to a clock.

All the tests were run by one examiner only with experience in scholar revisions after a previous calibration (Friedman’s test) for both dental measurements and lateralities with a senior examiner.

Statistic Analysis: The mean of each one of the parameters (dentition, hand, foot, eye and ear) was calculated. All the results were stored in a Excel (Microsoft Office®) program database and the statistic analysis were done by the program Statgraphics Plus 5.1 (Statistical Graphics Corp. 2001).

Different levels of statistical analysis were considered: an univariate study to assess the percentage of right laterality and left laterality for each one of the variables and a bivariate study to assess the relationship between each one of the other lateralities and dentition. Then 2x2 table was built showing the frequency of the occurrence for the two compared values at every moment in time. After that it was made a multivariate study to carry out a logistic regression taking the dependent variable as reference. The prevalence of right hand laterality in the studied population was considered as 0,9 (90%) (12). Sensitivity, specificity, positive predictive value and negative predictive value of foot laterality and dentition as diagnostic tests of hand laterality were evaluated. In last place, hand laterality-dentition laterality variable relation and hand laterality-feet laterality relation, as a function of sex were studied.

## Results

In the univariate study, it was accounted the number of times that each of the dichotomic values (right or left) occurred on dentition, hand, foot, eye and hearing lateralities. It can be observed a higher percentage of right laterality in each one of the parameters measured. About the relationship between dental eruption laterality and the other lateralities respectively (hand, foot, eye, and hearing), the chi-square test applied showed a *p*-value of < 0,0001. Consequently, the observed dentition laterality value for a particular case was related to laterality value for each one of the other variables studied (hand, foot, eye, hearing).

In the logistic regression based on the hand laterality, both foot and dentition laterality behaved as the only independent variables, while eye and hearing lateralities lost their statistic significance at interacting with the others ( Table 1).

**Table 1:**
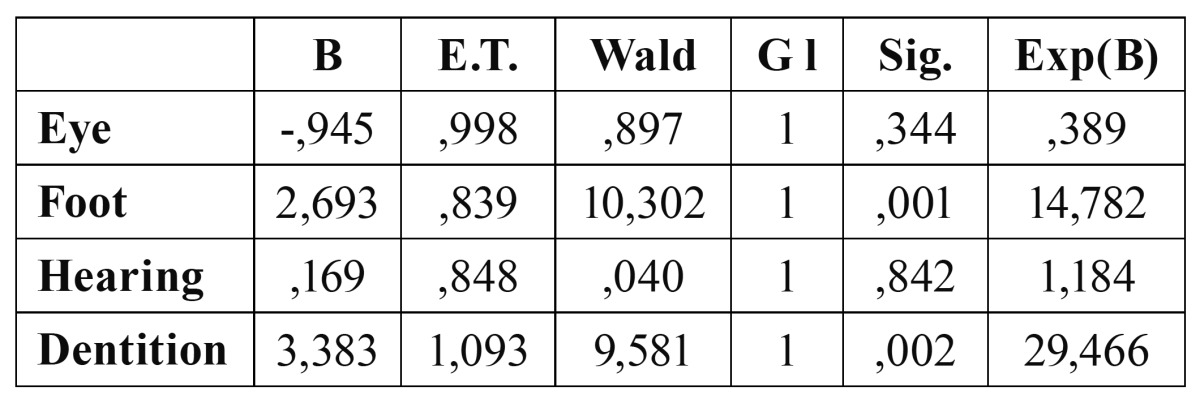
Logistic regression based on the hand laterality, Variables on the equation (Variables introduced in step one: eye, foot, hearing and dentition).

The results of the study of sensibility, specificity, positive predictive value and negative predictive value on the obtained data were graphed too ( Table 2, Table 3).

**Table 2:**
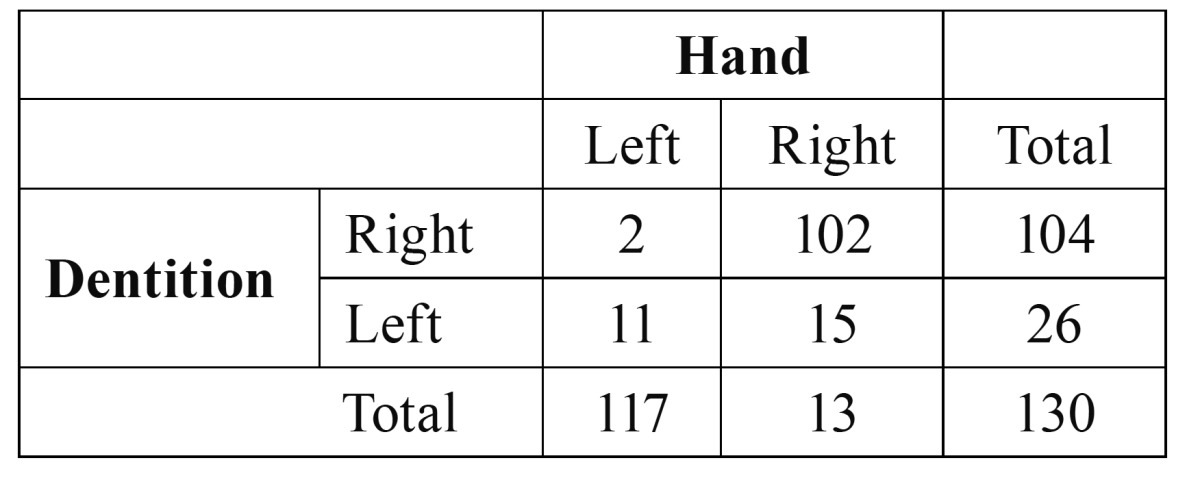
Contingency for mouth-hand.

**Table 3:**
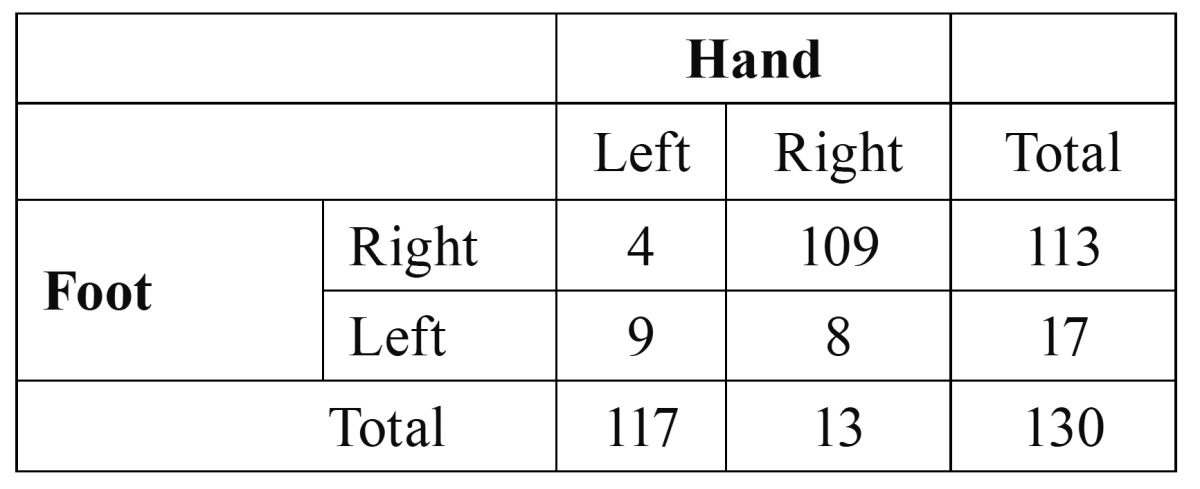
Contingency for foot-hand.

Mouth-hand:

• Sensibility: 102 / 117 = 87%.

• Specificity: 11 / 13 = 84,6%

• Positive predictive value: 102 / 104 = 98%

• Negative predictive value: 11 /26 = 42,3 %

Foot-hand:

• Sensibility: 109 / 117 = 93,2%

• Specificity: 9 / 13 = 69,2%

• Positive predictive value: 109 / 113 = 96%

• Negative predictive value: 9 /17 = 53 % 

From the analysis of relationships hand-dentition laterality and hand-foot laterality as a function of sex, crossed laterality hand-foot is significantly more predominant in men (13%) than in women (1,6%). Meanwhile, the relationship between hand and dentition didn’t show any influence of sex.

## Discussion

The aim of the study was to compare relationship between laterality of dental eruption and motor lateralities in 130 children belonging to a multiethnic primary education area of Barcelona with a mean age of 6,723 years.

-Discussion of the methodology of the study

The study has been designed as a transversal one with conglomerates (all the class members of schools from the selected area). Although it is not a random sample study, the final results could represent the reality of a children population between 6 and 8 years. As the examination included all the members of the sample, extrapolation of the results of this study to the rest of the population of these ages could be feasible as representative ones.

Because of final data were collected by a single observer there aren’t any dependent variables aroused from an evaluation performed by different operators; therefore the values taken in our measurements (after repeated them and found no significative differences) can be considered reliable and comparable between them.

In other studies previously made on this topic, one limitation lies in the value for the dental eruption order variable that was collected by indirect way (parent questioning), so the data were based on memories, photographs, registrations and recordings, providing not completely reliable information (6,7). Another difference lies in the fact that hand laterality in those studies was evaluated in 3 to 5 years children, but that period could be precocious or premature as it has been considered that in these age span would not yet set a complete maturity in that domain (1).

In our case, the tests applied for the lateralities had been set using together all the parameters proposed by different specialists in child psychology and pedagogy .This way, we evaluate the maximum number of values used by them to obtain more reliability and reproducibility in ours tests. As far as the test of dental eruption laterality is concerned, it was studied the dentition (type of dentition and order of eruption) comparing left and right hemiarcades first definitive molars levels and superior and inferior incisives (7).

-Discussion of the results 

Although the relationship between eruption pattern and motor laterality was previously described, our study attemps to provide a more thoroughly information regarding the possible role of the dental eruption pattern as a motor laterality indicator. Particularly when we consider that it has been studied a larger number of individuals, it has been used a full set of statistical tests and it has been evaluated exhaustively the pattern of teeth eruption, something that was not done before.

Results on the relationship between laterality and dental eruption are essentially the same to those of similar studies (6,7). In those, even if the evaluation of dentition was not as exhaustive, it was observed that dental eruption might be a good indicator of a child laterality. Both univariant and bivariant studies showed statistically significant results at all the studied variables: *p*-value > 0,01. In bivariant studies, the observed dentition laterality value for a particular case could be related with its value in each of the others studied lateralities (hand, feet, eye and hearing). But in logistic regression we found that the eye and ear laterality variables lost their statistical significance by interacting with the previous ones (13). Thus, only foot and dentition lateralities behave as independent variables which could foretell hand latera-lity. This means not only that dentition laterality (along with the foot one) would be one of the parameters more related to the hand laterality, but also as dental eruption variable (expB) presented a greater relationship than the foot one. According with this results it should suggest that dental eruption could be more clinically relevant. As far as the validity of diagnostic tests are evaluated by sensibility and specificity indexes, our results showed that both dentition and foot lateralities present a high sensibility but dentition one showed greater specificity values. That could make tooth eruption laterality, again, a more reliable parameter to determine the true child laterality and would provide a greater diagnosis relevance.

Different predictive values and efficiency of the test were calculated in order to assure the behavior of our variable when it was used in different clinical contexts. Dentition laterality, as well as foot laterality, showed a high positive predictive value, however neither of them show a relevant negative predictive value.

From the analysis of the hand-dentition and hand-foot laterality relationships as a function of gender, it should be remarked the fact that crossed laterality (hand-foot) was more prevalent among men (13%) than women (1,6%). Meanwhile hand-dentition relationship didn’t seem to be affected by gender. This fact would provide clinical usefulness as laterality in dentition could be used as predictive factor of general laterality (hand as reference) independently of gender.

Consequently, according to the results obtained, our study should suggest that dental eruption laterality could be used as another parameter in the determination of a patient’s laterality as the predominant side of dental eruption order was related to laterality observed in the other four parameters studied (6,7). Although dental eruption and foot laterality variables presented a strong connection with hand laterality, dentition was the one that would offer a higher diagnostic and prognostic relevance. Thus, dental eruption predominant side order would seem to be the best parameter to predict one’s laterality alongside the hand as dental laterality showed a great specificity and no significant gender differences. So it could be possible to use it to help to establish the true laterality of a child with an undefined or unclear one and also could help to precise the predominant laterality in a crossed laterality child. Thus, it would be useful to work with paediatricians, educators and parents about the value of dental eruption as a prognostic test for the hand laterality.
